# A double bond with weak σ- and strong π-interactions is still a double bond

**DOI:** 10.1038/s41467-021-24238-x

**Published:** 2021-06-29

**Authors:** Cina Foroutan-Nejad

**Affiliations:** Institute of Organic Chemistry, Polish Academy of Sciences, Warsaw, Poland

**Keywords:** Chemical bonding, Computational chemistry

Arising from Kyushin et al. *Nature Communications* 10.1038/s41467-020-17815-z (2020)

Synthesis of 1,3 singlet diradicals in cyclic organic molecules has been the main route toward the formation of single π bonds, i.e., π-bonded atoms without σ-bonding^1–4^ by forming a long-range π-bond between unpaired electrons of the radicals that occupy *p*-atomic orbitals. In a recent study, Kyushin et al. reported synthesis and characterization of 1,2,2,3,4,4-hexa-*tert*-butylbicyclo[1.1.0]tetrasilane (**2**), a four-membered singlet diradical silicon ring with a 1,3 silicon-silicon single π-bond and identified a single π-bond by analyses based on canonical molecular orbitals (CMOs) and natural bond orbitals^5^ (NBOs). Although MO-based analyses confirm that the two *sp*^*2*^ hybridized silicon atoms are connected via a π-MO, the HOMO of the molecule^4^, a deep analysis on the basis of topological approaches indicates a different story. Here, I study this system in more details and show that this bond is not a single π-bond, but rather a unique double bond in which the role of σ- and π bonds is reversed, i.e., a weak σ-bond and a strong π-bond.

The first evidence in favor of σ-bonding can be found in CMOs, where HOMO −6 of the molecule represents a σ-interaction between two *sp*^*2*^ hybridized silicon atoms, Fig. 1. This orbital with HOMO −1, HOMO −2, and HOMO −3 constitute the sigma framework of the silicon ring. Kyushin et al. in their contribution suggest that HOMO −1 is an antibonding orbital that quenches the bonding effect of HOMO −6. However, MO-based analyses do not provide a quantitative picture of bonding^6^. MOs change shape by choosing different isosurface values or by employing various types of orbitals. Besides, in polyatomic molecules, the role of bonding and antibonding MOs is not comparable with simple diatomics, where a qualitative analysis can explain the bonding characters of a system.

**Fig. 1:**
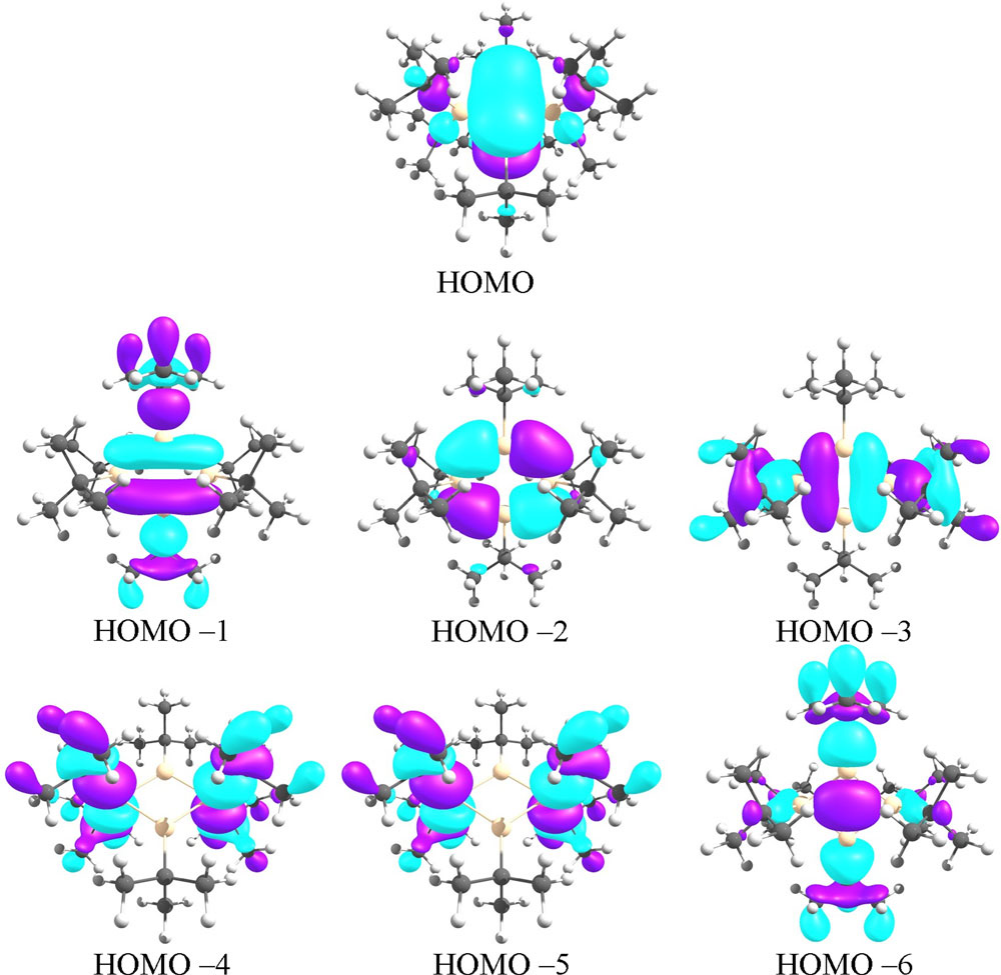
Occupied molecular orbitals. Occupied molecular orbitals of **2**; HOMO −6 corresponds to a σ-bond across the ring.

To obtain a quantitative picture of the bonding in **2**, the molecule was analyzed within the context of Bader’s theory^7^. Topological analysis of the electron density identifies no bond critical point (BCP) between the two *sp*^*2*^ hybridized silicon atoms. Nevertheless, the presence or absence of BCPs is not a reliable measure of bonding; therefore, other parameters were studied^8^. Ideally a pure π-bond should have 2 BCPs above and below the nodal plane of the π-MO; however, this feature is visible only if the σ-electron density is removed^9,10^. In ordinary molecules with double bonds, the core and σ-electrons mask the fingerprint of π-electron density. To visually search for a potential single π-bond, the derivatives of the electron density, namely the Laplacian of the electron density, ${\nabla }^{2}\rho (r)$, and energy density were probed.

The plot of ${\nabla }^{2}\rho (r)$ shows regions of electron density concentration on Si atoms corresponding to their atomic *p* orbitals, Fig. 2a. This feature becomes more pronounced after removing electrons from the σ-skeleton but the region in between two Si atoms has a positive Laplacian that is a feature of noncovalent interactions or that of charge-shift bonds^7,11^. The Laplacian in the central region of the ring remains positive after removing electrons from the σ-framework of the Si_4_ ring, Fig. 1b, c. Although no BCP between two Si atoms is found, some of the properties of the ring critical point (RCP) in the middle of the Si_4_ ring are interesting. The electron density of this point is 0.0311 *e*.${a}_{0}^{-3}$. The contribution of HOMO −6, the bonding σ-MO, to the local electron density to is 0.0086 *e*.${a}_{0}^{-3}$, comparable with that of a weak hydrogen bond^12^. The sum of the core electrons of the Si atoms and valance electrons of the other atoms constitute the rest of the 0.0225 *e*.${a}_{0}^{-3}$ of the electron density of the RCP. It is worth emphasizing that at the RCP the π-electron density and contributions of the other σ-MOs equals zero because the RCP falls on the nodal plane of these orbitals. Removing electrons from HOMO −6 shifts ${\nabla }^{2}\rho \left(r\right)$ of the RCP from 0.0654 *e*.${a}_{0}^{-5}$. to a more positive value, 0.0735 *e*.${a}_{0}^{-5}$ because the negative eigenvalue of the Hessian of the electron density, λ_1_, which reflects the out-of-plane curvature of the local electron density, changes from −0.0111 *e*.${a}_{0}^{-5}$ to −0.0036 *e*.${a}_{0}^{-5}$ when electrons of HOMO −6 are removed from the total electron density. This change suggests that HOMO −6 has the role of a bonding MO in between 1, 3 Si atoms.

**Fig. 2:**
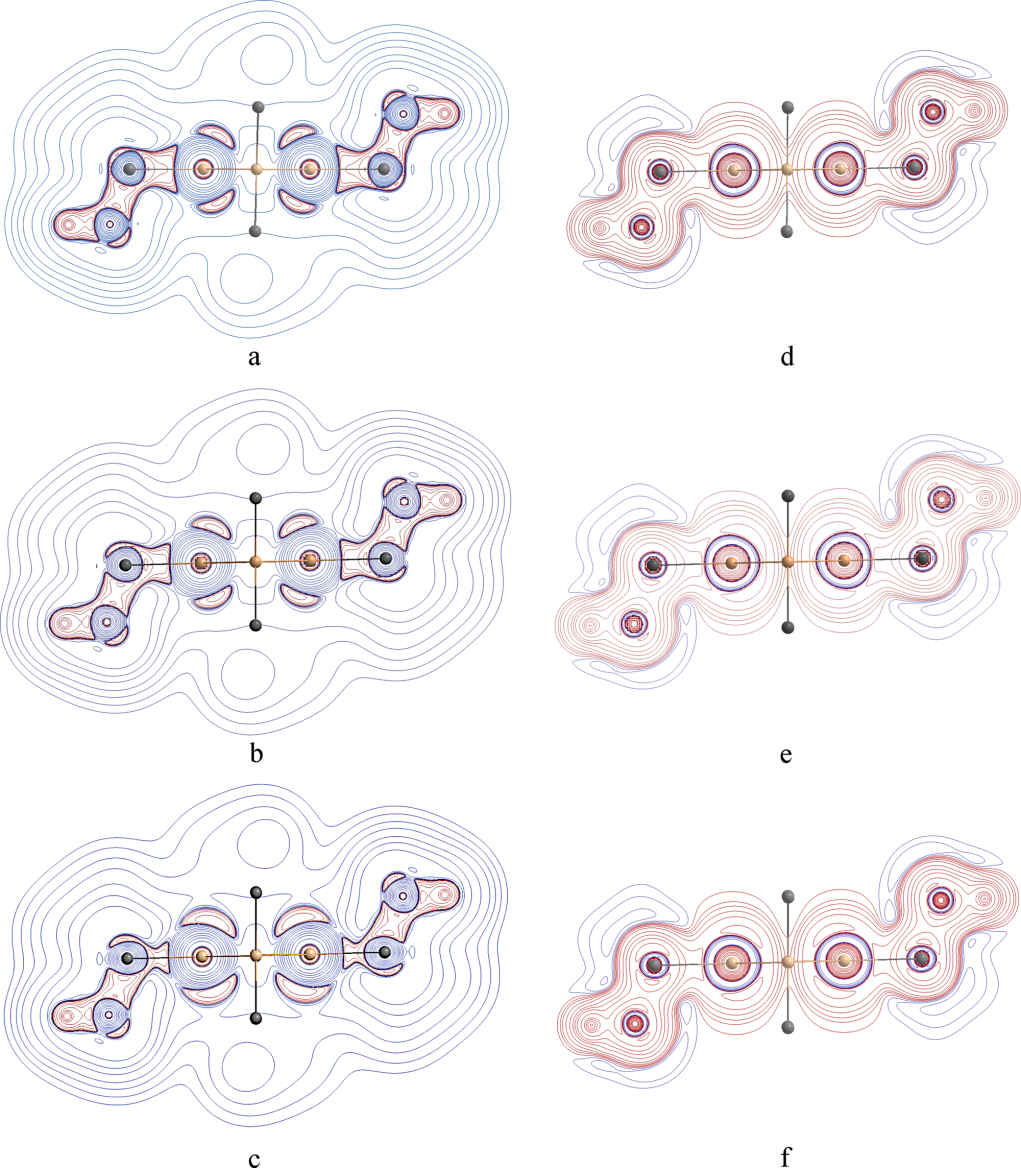
Topology of the derivitives of the electron density. Topology of the derivitives of the electron density; contour plots of the Laplacian of electron density (**a**–**c**) and energy density (**d**–**f**) in **2**. CH_3_ groups are removed for more clarity in the plot. Positive and negative values are represented by blue and red lines, respectively. **a** The central region between 1,3 atoms has a positive Laplacian that is a sign of closed-shell interaction. **b** By removing electrons from the σ-bonding HOMO −6 the electron density concentrations, corresponding to the *p-*atomic orbitals on the Si atoms intensifies. **c** Removing all electrons from the σ-framework of Si_4_ intensifies the *p*-electron density concentration. **d** Contour plot of energy density that shows a covalent-type interaction between Si atoms. **e** Contour plot of energy density after removing electrons from HOMO −6, and (**f**) after removing electrons from all σ-MOs of Si_4_ moiety. The p-atomic orbital-shaped feature appears on the Si atoms by removing the σ-electrons in the contour plot of energy density too.

The contour plots of the energy density are consistent with covalent-type interaction between the Si atoms because the energy density remains negative in the mid-ring region, Fig. 2d–f. Removing the σ-electrons from HOMO −6 changes the distribution of the energy density and reduces its absolute value at the center of the ring from −0.0028 *E*_*h*_.${a}_{0}^{-3}$ to −0.0008 *E*_*h*_.${a}_{0}^{-3}$, reemphasizing the bonding contribution from HOMO −6. Removing the rest of σ-electrons does not change the energy density or Laplacian at the RCP but intensifies the features of π bond by forming some local maxima at the position of *p*-atomic orbitals corresponding to the negative region of Laplacian on the Si atoms as it is evident in Fig. 2e, f.

The most reliable piece of information regarding the contribution of the π- and σ-frameworks to the bonding can be obtained from computing the delocalization index (DI), a direct measure of covalency, between 1,3 Si atoms^13–15^. The total DI between the atoms is 0.73 that is comparable with the DI of a single bond (~1.0) in a homonuclear species. The individual contributions of the π- and σ-electrons in the DI are 0.51 and 0.22, respectively. These values suggest that although a weak π-interaction forms between 1,3 silicon atoms, a weaker but nonnegligible σ-interaction is present between the same pair of atoms.

In a nutshell, **2** is a unique system in which π-bond is stronger than the σ-bond but it still is a double-bonded system. In my humble opinion, synthesis of a system with reversed π- and σ-bonding strengths, is more notable than the synthesis of a pure π-bond because examples of the latter are known^16–19^, but the former is a lesser-known phenomenon.

## Methods

The structure of the molecule was taken from the Supplementary Information of the original paper by Kyushin et al.^4^ and reoptimized at two levels of theory, B3LYP/6-31g(d) as the original paper (Table S1) and B3LYP/def2-TZVP (Table S2) for more accuracy by Gaussian 16^20^. No symmetry was imposed during optimization. A local minimum with lower energy compared to the previously reported structure was found and confirmed via frequency computations. The stability of the wavefunction was examined and the system was found to be stable at the selected computational level. The Si_4_ framework keeps its planar structure with D_*2h*_ symmetry in the new conformer that allows one to separate σ- and π-frameworks by removing electrons from the corresponding MOs within the framework of Bader’s theory. CMOs of the molecule were analyzed at both levels of theory and no difference was found. The wavefunctions were analyzed and visualized by AIMAll^21^ at both levels of theory. The values reported here are from B3LYP/6-31g(d) akin to those reported in the original paper. To evaluate the effect of each MO on bond characteristics, occupation of the corresponding MO in the WFX files was nullified and the bonding analyses repeated. The difference between the magnitude of each parameter in the original wavefunction and the manipulated one provides the effect of the manually unoccupied MO on the desired properties. To compute individual π- and σ-contributions to the DI, the occupation number of the π-MO was nullified. It is worth noting that removing electrons from the σ-framework is not recommended because the σ-electrons constitute the backbone of the electron density and removing them can change the topology of atoms and affect the magnitude of DI^14,22^.

## Supplementary information

Supplementary Information

## Data Availability

The optimized structure of **2** at two levels of theory are provided in the Supplementary Information.
