# Supplementary material for: A double bond with weak σ- and strong π-interactions is still a double bond
Source: Nat Commun. 2021 Jun 29;12:4037. doi: 10.1038/s41467-021-24238-x (PMC8241832; doi:10.1038/s41467-021-24238-x)
Supplement: Supplementary file 1 — Supplementary Information [file 41467_2021_24238_MOESM1_ESM.pdf]

Supplementary Information

A Double Bond with Weak σ- and Strong π-Interactions Is Still a Double Bond

Cina Foroutan-Nejad[1]*

[1]Institute of Organic Chemistry, Polish Academy of Sciences, Kasprzaka44/52, 01-224 Warsaw, Poland

Correspondence: cforoutan-nejad@icho.edu.pl

Supplementary Table 1.
The cartesian coordinate of the optimized structure at B3LYP/6-31g(d) level

Energy: **-2105.04182915 au**

symmetry c1

Si	0.094476000	-1.434119000	-0.362267000
Si	-1.057492000	-1.808565000	1.623647000
Si	-2.593071000	-0.800446000	0.196654000
Si	-1.441107000	-0.425943000	-1.789246000
C	1.965691000	-1.867843000	-0.747298000
C	2.865775000	-0.674885000	-0.355504000
C	2.398176000	-3.107733000	0.060356000
C	2.144161000	-2.157196000	-2.250917000
C	-4.464319000	-0.366855000	0.581655000
C	-5.364342000	-1.559774000	0.189607000
C	-4.896776000	0.873166000	-0.225815000
C	-4.642879000	-0.077766000	2.085315000
C	-0.444854000	-0.750548000	3.175320000
C	-1.444257000	-3.687795000	2.096028000
C	-1.054336000	1.453275000	-2.261599000
C	-2.053801000	-1.483935000	-3.340903000
C	-0.505627000	0.750349000	2.831237000
C	-1.315825000	-0.985480000	4.428360000
C	1.022961000	-1.090766000	3.519507000
C	-1.552940000	-4.528452000	0.809131000
C	-0.342998000	-4.313002000	2.979139000
C	-2.795786000	-3.793568000	2.838051000
C	-0.945716000	2.293933000	-0.974698000
C	0.297194000	1.559091000	-3.003597000
C	-2.155575000	2.078482000	-3.144751000
C	-1.993190000	-2.984833000	-2.996779000
C	-3.521563000	-1.143605000	-3.685211000
C	-1.182717000	-1.249127000	-4.593897000
H	3.919404000	-0.911896000	-0.569383000
H	2.794659000	-0.437074000	0.712054000
H	2.614836000	0.233091000	-0.915748000
H	3.453866000	-3.343071000	-0.142461000
H	1.808484000	-3.994063000	-0.202160000
H	2.301068000	-2.947510000	1.140254000
H	3.197925000	-2.386076000	-2.470449000
H	1.860862000	-1.298290000	-2.869972000
H	1.547292000	-3.017438000	-2.576368000
H	-6.417991000	-1.322839000	0.403474000
H	-5.113396000	-2.467839000	0.749707000
H	-5.293164000	-1.797399000	-0.877989000
H	-5.952482000	1.108456000	-0.023027000

H	-4.799600000	0.713123000	-1.305734000
H	-4.307112000	1.759458000	0.036887000
H	-5.696664000	0.151030000	2.304831000
H	-4.046063000	0.782446000	2.410943000
H	-4.359570000	-0.936767000	2.704234000
H	-0.146389000	1.343433000	3.685565000
H	-1.525596000	1.081087000	2.601962000
H	0.118049000	0.999598000	1.966113000
H	-2.372052000	-0.755407000	4.245691000
H	-0.977719000	-0.331566000	5.246158000
H	-1.253704000	-2.014497000	4.796341000
H	1.365822000	-0.470449000	4.361359000
H	1.692792000	-0.888037000	2.675659000
H	1.159802000	-2.136358000	3.811468000
H	-1.794150000	-5.571318000	1.063949000
H	-0.616405000	-4.533256000	0.238925000
H	-2.337164000	-4.157649000	0.140687000
H	-0.567837000	-5.374734000	3.160460000
H	-0.266397000	-3.831323000	3.959013000
H	0.643391000	-4.267846000	2.502804000
H	-3.016851000	-4.846393000	3.069974000
H	-3.623161000	-3.418002000	2.224688000
H	-2.804941000	-3.244814000	3.784719000
H	-0.704513000	3.336802000	-1.229507000
H	-0.161508000	1.923146000	-0.306226000
H	-1.882275000	2.298715000	-0.404532000
H	0.518214000	2.611923000	-3.235529000
H	0.306381000	1.010325000	-3.950257000
H	1.124578000	1.183565000	-2.390222000
H	-1.930723000	3.140211000	-3.326068000
H	-3.141978000	2.033337000	-2.668441000
H	-2.232146000	1.596796000	-4.124622000
H	-2.352444000	-3.577901000	-3.851110000
H	-2.616929000	-3.234006000	-2.131677000
H	-0.973264000	-3.315663000	-2.767447000
H	-3.864415000	-1.763930000	-4.527059000
H	-3.658311000	-0.098017000	-3.977221000
H	-4.191466000	-1.346246000	-2.841398000
H	-1.520789000	-1.903078000	-5.411679000
H	-0.126516000	-1.479234000	-4.411134000
H	-1.244767000	-0.220130000	-4.961949000

Supplementary Table 2.
The cartesian coordinate of the optimized structure at B3LYP/def2-TZVP level

Energy: **-2105.49436258 au**

symmetry c1

Si	0.074962000	-1.435649000	-0.360696000
Si	-1.057181000	-1.809265000	1.625845000
Si	-2.573579000	-0.798915000	0.195097000
Si	-1.441432000	-0.425281000	-1.791437000
C	1.939499000	-1.864163000	-0.743087000
C	2.829479000	-0.669993000	-0.347991000
C	2.377327000	-3.099018000	0.060299000
C	2.125123000	-2.149380000	-2.241740000
C	-4.438133000	-0.370464000	0.577472000
C	-5.328065000	-1.564718000	0.182525000
C	-4.876034000	0.864287000	-0.226036000
C	-4.623738000	-0.085094000	2.076098000
C	-0.445678000	-0.757776000	3.175201000
C	-1.442982000	-3.682005000	2.101203000
C	-1.055650000	1.447458000	-2.266807000
C	-2.052931000	-1.476765000	-3.340797000
C	-0.500617000	0.738922000	2.831330000
C	-1.315136000	-0.987342000	4.424930000
C	1.016298000	-1.098723000	3.524667000
C	-1.557671000	-4.520486000	0.818637000
C	-0.344036000	-4.309016000	2.978295000
C	-2.787587000	-3.790055000	2.846759000
C	-0.940722000	2.285902000	-0.984238000
C	0.288831000	1.555481000	-3.012590000
C	-2.154729000	2.074520000	-3.143695000
C	-1.998312000	-2.973451000	-2.996821000
C	-3.514798000	-1.135563000	-3.690470000
C	-1.183273000	-1.247440000	-4.590431000
H	3.879977000	-0.903157000	-0.560059000
H	2.755898000	-0.435626000	0.715487000
H	2.577101000	0.233818000	-0.905476000
H	3.431023000	-3.323909000	-0.142084000
H	1.797386000	-3.985479000	-0.204937000
H	2.278110000	-2.943047000	1.135871000
H	3.177652000	-2.370936000	-2.453673000
H	1.841342000	-1.295121000	-2.858740000
H	1.537970000	-3.009767000	-2.569495000
H	-6.378569000	-1.331584000	0.394596000
H	-5.075628000	-2.468458000	0.740098000
H	-5.254500000	-1.799194000	-0.880929000
H	-5.929737000	1.089150000	-0.023657000

H	-4.776831000	0.708208000	-1.301593000
H	-4.296130000	1.750803000	0.039095000
H	-5.676271000	0.136444000	2.288029000
H	-4.036611000	0.775350000	2.403749000
H	-4.339912000	-0.939276000	2.693183000
H	-0.141080000	1.325600000	3.684535000
H	-1.514587000	1.072991000	2.602532000
H	0.122308000	0.985067000	1.970711000
H	-2.366376000	-0.754818000	4.244178000
H	-0.973600000	-0.333806000	5.236053000
H	-1.255294000	-2.011132000	4.794197000
H	1.351403000	-0.477556000	4.363608000
H	1.687990000	-0.898456000	2.688004000
H	1.150212000	-2.139194000	3.819009000
H	-1.798972000	-5.557879000	1.077509000
H	-0.627377000	-4.528746000	0.247217000
H	-2.340384000	-4.150548000	0.155637000
H	-0.573554000	-5.366098000	3.155955000
H	-0.264971000	-3.831742000	3.954984000
H	0.637529000	-4.267537000	2.502229000
H	-3.001713000	-4.840816000	3.074911000
H	-3.615373000	-3.417788000	2.240583000
H	-2.793521000	-3.246208000	3.790943000
H	-0.699434000	3.323296000	-1.243124000
H	-0.157907000	1.915926000	-0.321380000
H	-1.870920000	2.294175000	-0.412662000
H	0.502953000	2.606239000	-3.240757000
H	0.294584000	1.011652000	-3.956786000
H	1.116709000	1.183173000	-2.406562000
H	-1.925189000	3.131589000	-3.321409000
H	-3.136205000	2.033098000	-2.667440000
H	-2.234009000	1.597242000	-4.120365000
H	-2.357862000	-3.560117000	-3.850029000
H	-2.621381000	-3.219424000	-2.136257000
H	-0.984430000	-3.307695000	-2.767887000
H	-3.849907000	-1.756703000	-4.529428000
H	-3.648477000	-0.095079000	-3.984875000
H	-4.186637000	-1.335668000	-2.853887000
H	-1.524848000	-1.900941000	-5.401565000
H	-0.132105000	-1.480179000	-4.409540000
H	-1.242851000	-0.223655000	-4.959753000
